# Metabolic engineering of *Escherichia coli* W for isobutanol production on chemically defined medium and cheese whey as alternative raw material

**DOI:** 10.1007/s10295-020-02319-y

**Published:** 2020-10-17

**Authors:** Katharina Novak, Juliane Baar, Philipp Freitag, Stefan Pflügl

**Affiliations:** Institute for Chemical, Environmental and Bioscience Engineering, Research Area Biochemical Engineering, Technische Universität Wien, Gumpendorfer Straße 1a, 1060 Vienna, Austria

**Keywords:** Chemically defined medium, Promotor fine-tuning, Constitutive promotor, Pulsed fed-batch, Isobutanol adaptation

## Abstract

The aim of this study was to establish isobutanol production on chemically defined medium in *Escherichia coli*. By individually expressing each gene of the pathway, we constructed a plasmid library for isobutanol production. Strain screening on chemically defined medium showed successful production in the robust *E. coli* W strain, and expression vector IB 4 was selected as the most promising construct due to its high isobutanol yields and efficient substrate uptake. The investigation of different aeration strategies in combination with strain improvement and the implementation of a pulsed fed-batch were key for the development of an efficient production process. *E. coli* W *ΔldhA ΔadhE Δpta ΔfrdA* enabled aerobic isobutanol production at 38% of the theoretical maximum. Use of cheese whey as raw material resulted in longer process stability, which allowed production of 20 g l^−1^ isobutanol. Demonstrating isobutanol production on both chemically defined medium and a residual waste stream, this study provides valuable information for further development of industrially relevant isobutanol production processes.

**Electronic supplementary material:**

The online version of this article (10.1007/s10295-020-02319-y) contains supplementary material, which is available to authorized users.

## Introduction

Second-generation biofuels, which are produced from lignocellulosic biomass or waste streams, are considered as strategically important sustainable fuels due to their renewability, biodegradability and low emissions of greenhouse gases [1]. The production of higher molecular weight alcohols such as n-butanol and isobutanol poses advantages over ethanol production. Both alcohols have a higher energy content closer to gasoline, can be transported using existing infrastructure and their lower vapor pressures improves mixing with gasoline [2, 3]. Isobutanol has a higher octane number than n-butanol, is less toxic to cells and requires less energy for downstream processing [2]. Biotechnologically, the isobutanol pathway is less complex and not acetyl-CoA dependent, which results in lower side-product formation compared to n-butanol production [4].

Isobutanol is a metabolite not naturally synthesized by any organism. However, its synthesis is possible through a combination of the valine biosynthesis and the Ehrlich pathway [5]. The production of isobutanol has been demonstrated in several organisms, including *Escherichia coli* [5, 6], *Saccharomyces cerevisiae* [7, 8], *Corynebacterium glutamicum* [9], *Clostridium thermocellum* [10] and even autotrophic organisms like *Ralstonia eutropha* [11] and *Synecococcus elongatus* [12]. Advances in microbial isobutanol production have recently been reviewed [4].

**Fig. 1:**
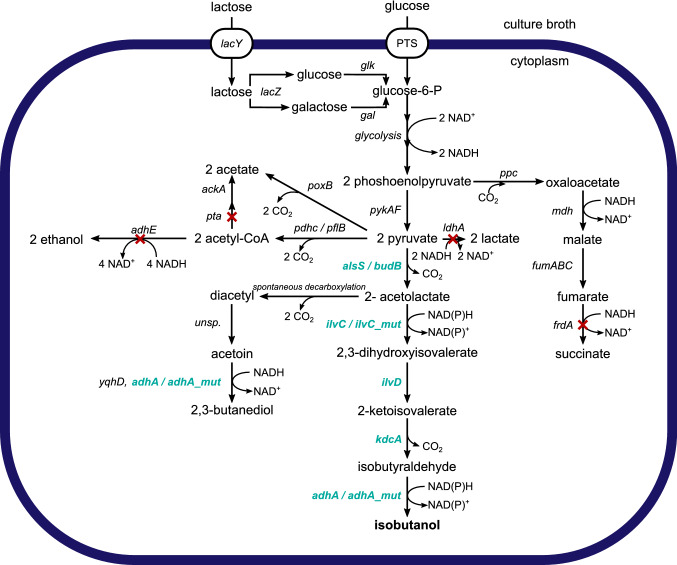
Metabolic network of *Escherichia coli* for isobutanol production, substrate uptake and by-product formation. Bold green genes were overexpressed. Red crosses indicate deleted genes in *E. coli* W *∆ldhA ∆adhE ∆pta ∆frdA*. *Unsp*. indicates unspecific reactions (color figure online)

Synthesis of isobutanol from pyruvate relies on a five-enzyme pathway (Fig. 1). Acetolactate synthase (AlsS or BudB) converts two molecules of pyruvate to 2-acetolactate, which is further processed to 2,3-dihydroxyvalerate by ketol-acid reductoisomerase (IlvC). Dihydroxy-acid dehydratase (IlvD) catalyzes the reaction to 2-ketoisovalerate and α-ketoisovalerate decarboxylase (KdcA) converts it to isobutyraldehyde. Finally, isobutanol is produced by an alcohol dehydrogenase (AdhA). Two of these enzymes, *ilvC* and *ilvD*, are native to *E. coli* [5]. Acetolactate synthase from *Bacillus subtilis* (AlsS) was used for efficient isobutanol production [13]. Acetolactate synthetase is also the first enzyme in the 2,3-butanediol production pathway of natural producers. For example, *budB* codes for acetolactate synthase from *Enterobacter cloacae* subsp*. dissolvens* [14, 15]. *E. coli* cannot naturally produce isobutanol because it lacks an α-ketoisovalerate decarboxylase [16]. Overexpression of α-ketoisovalerate decarboxylase (*kdcA)* from *Lactococcus lactis* was shown to result in high isobutanol yields [13]. *E. coli* has a native alcohol dehydrogenase *yqhD*, but overexpression of *adhA* from *L. lactis* is advantageous due to utilization of NADH rather than NADPH as a cofactor [17]. A mutant of *adhA* was shown to have higher affinity towards isobutyraldehyde. In a mutated form of *ilvC*, the cofactor was exchanged from NADPH to NADH. These mutations enabled anaerobic isobutanol production at 100% of the theoretical yield [18].

Since isobutanol is toxic to microorganisms [3], procedures for product removal from the culture broth are important to obtain high titers [19]. Until now, the highest isobutanol titer of 50 g l^−1^ was achieved in a fed-batch fermentation of *Escherichia coli* applying in situ product removal by gas stripping [6]. The elimination of competing pathways (Fig. 1) for acetate, lactate, succinate and ethanol formation was shown to increase isobutanol yields and thus enabled production at higher titers [5, 6]. However, production was only achieved by the addition of complex media components such as yeast extract (Table 1). When defined medium is used, all cellular components must be synthesized de novo, whereas different precursors can be metabolized when complex media components such as yeast extract are added. The drawback and limitations of the use of complex media components have recently been intensively investigated and described [20]. In short, utilization of yeast extract modulates growth parameters like the specific growth rate µ due to the depletion of components throughout the cultivation [20, 21]. It leads to a lack in reproducibility due to variable composition [22] and to differences in cellular metabolism, e.g., in acetate excretion and protein expression [23].

**Table 1:** State-of-the-art of heterotrophic isobutanol production processes and their performance parameters as described in literature

Host organism	Substrate	Complex media additives	Overexpressed genes	Host engineering	Titer	Isobutanol production rate	% of max. theor. yield	Production system	References
*Escherichia coli*	Glucose	None	*budB, ilvC_mut, ilvD, kdcA, adhA_mut*	*ΔldhA ΔadhE Δpta ΔfrdA*	16 g l^−1^	0.25 g l^−1^ h^−1^	38%	Fed-batch (aerobic)	This study
*Escherichia coli*	Cheese whey	None	*budB, ilvC_mut, ilvD, kdcA, adhA_mut*	*ΔldhA ΔadhE Δpta ΔfrdA*	20 g l^−1^	0.29 g l^−1^ h^−1^	39%	Fed-batch (aerobic)	This study
*Escherichia coli*	Glucose	5 g l^−1^ yeast extract	*alsS, ilvCD, kivd, adhA*	*∆adhE, ∆frdBC, ∆fnr-ldhA, ∆pta,* *∆pflB*	22 g l^−1^		86%	Shake flasks (microaerobic)	[5]
*Escherichia coli*	Glucose	25 g l^−1^ yeast extract	*alsS, ilvCD, kivd, adhA*	*∆adhE, ∆frdBC, ∆fnr-ldhA, ∆pta,* *∆pflB*	50 g l^−1^	0.7 g l^−1^ h^−1^	86%	Fed-batch (aerobic)	[6]
*Escherichia coli*	Glucose	10 g l^−1^ yeast extract	*alsS, ilvD, kivD, ilvC_mut, adhA_mut*	*ΔldhA, ΔadhE, Δfrd, ΔpflB, ΔilvC, Δpta*	13.4 g l^−1^	0.09 g l^−1^ h^−1^ OD^−1^	103%	Bottles (anaerobic)	[18]
*Escherichia coli*	Cellobiose	5 g l^−1^ yeast extract	*alsS, ilvCD, kivD, adhA, bglC*	*∆adhE, ∆frdBC, ∆fnr-ldhA, ∆pta,* *∆pflB*	7.6 g l^−1^	0.16 g l^−1^ h^−1^	28%	Shake flasks	[37]
*Escherichia coli*	Glucose and xylose	5 g l^−1^ yeast extract	*alsS, adhA, kivD, ilvCD*	*ΔldhA ΔadhE ΔpflB ΔackA-pta*	8.4 g l^−1^	0.18 g l^−1^ h^−1^	66%	Shake flasks	[26]
*Escherichia coli*	Hydrolysate from cedar	None	*alsS, adhA, kivD, ilvCD*	*ΔldhA ΔadhE ΔpflB ΔackA-pta*	3.7 g l^−1^	0.04 g l^−1^ h^−1^	14%	Shake flasks	[26]
*Escherichia coli*	Sucrose	5 g l^−1^ yeast extract	*alsS, ilvC, ilvD, kivD*		1.7 g l^−1^		47%	Shake flasks	[25]
*Shimwellia blattae*	Wheat straw hydrolysate	5 g l^−1^ yeast extract	*alsS, ilvC, ilvD, kivD*		3 g l^−1^		25%	Shake flasks	[25]
*Corynebacterium glutamicum*	Glucose	5 g l^−1^ yeast extract	*ilvBNCD, kivd, adhA, pntAB*	*ΔaceE, Δpqo, ΔilvE, ΔldhA, Δmdh*	13 g l^−1^	0.33 g l^−1^ h^−1^	48%	Fed-batch (microaerobic)	[9]
*Saccharomyces cerevisiae*	Glucose	6.7 g l^−1^ yeast nitrogen base	*ILV2, ILV3, ILV5, KDC, ADH*	*ΔPDC1*	0.14 g l^−1^		1.6%	Shake flasks	[7]
*Saccharomyces cerevisiae*	Glucose	6.7 g l^−1^ yeast nitrogen base	*kivd, ADH6, ILV2, ILV5c, ILV3c, ILV2c, sMAE1*	*lpd1Δ*	1.6 g l^−1^		3.8%	Shake flasks	[8]
*Bacillus subtilis*	Glucose	5 g l^−1^ yeast extract, 10 g l^−1^ peptone	*alsS, ilvCD, kivd, adh2*		2.6 g l^−1^	0.09 g l^−1^ h^−1^		Microaerobic shake flask fed-batch	[38]
*Clostridium thermocellum*	Cellulose	4.5 g l^−1^ yeast extract	*kivd, ilvBN, ilvCD*	*Δhpt*	5.4 g l^−1^		41%	Bottles	[10]

Low material costs are pivotal for industrial production of cheap compounds such as fuel alcohols. The production cost of biobutanol has been reported to be greatly affected by the feedstock price, which accounts for 60–65% of the total production cost [24]. The cost of the production medium can be decreased by the omittance of expensive induction and complex media compounds as well as by the utilization of an alternative raw material. An overview over substrates that have been used for isobutanol production is depicted in Table 1. The use of lignocellulosic hydrolysates as cheap raw material has only led to the production of low amounts of isobutanol in *E. coli* and *Shimwellia blattae* [25, 26]. Lignocellulose is a promising raw material, but its pre-treatment poses some disadvantages such as high energy input and the accumulation of inhibitory side products [27]. In contrast, the use of cheese whey has been shown to increase growth rate, biomass yield and specific product titers during recombinant protein expression in *E. coli* [28]. In *Clostridium acetobutylicum*, the utilization of cheese whey enabled the production of 5.6 g l^−1^ butanol [29]. Annually, 180–190 million tons of cheese whey are produced worldwide during cheese or curd production [30]. High volumes and high organic content, mainly attributed to lactose, pose environmental burden on whey disposal [31]. To this end, microbial production of fuel chemicals from cheese whey could be a promising alternative for the reduction of media costs.

The aim of this study was to construct a recombinant *E. coli* strain for isobutanol production and establish an efficient, cheap, and easily feasible production process. The requirements to achieve this goal were defined as the following: (i) the use of constitutive promoters for fine-tuning of gene expression and to avoid the use of expensive inducers, (ii) the use of a robust *E. coli* strain with an optimized strain background, (iii) the improvement of titer, yield and production rate by the selection of appropriate process conditions, (iv) the use of defined medium to avoid expensive media components and (v) the utilization of cheese whey as an alternative raw material. Using this approach, a production system was established that produced isobutanol from glucose, lactose and cheese whey.

## Materials and methods

### Bacterial strains and media

For all general cloning steps and plasmid propagation, *Escherichia coli* BL21(DE3) (New England Biolabs, MA, USA) and *E. coli* Top 10 (kind gift of Prof. Michael Sauer, BOKU, Vienna, Austria) were used. *E. coli* W (DSM 1116 = ATCC 9637 = NCIMB 8666) from DSMZ (Braunschweig, Germany), *E. coli* W Δ*ldhA* Δ*adhE* Δ*pta* Δ*frdA* (kind gift of Prof. Michael Sauer, BOKU, Vienna, Austria) and *E. coli* K12-BW25113 (Yale CGSC, New Haven, US) were used for cultivations.

Lysogeny broth (LB) containing 10 g l^−1^ soy peptone, 5 g l^−1^ yeast extract and 10 g l^−1^ sodium chloride was used for all cloning and plasmid propagation steps. Soy peptone and yeast extract concentrations were doubled (giving 2x-LB) for bioreactor precultures. For cultivation on plates, 15 g l^−1^ agar was added to LB medium.

SOC medium containing 10 g l^−1^ NaCl, 2.5 mM KCl, 10 mM MgCl_2_, 10 mM MgSO_4_, 20 mM glucose, 20 g l^−1^ tryptone and 5 g l^−1^ yeast extract at pH 7.0 was used for transformant recovery.

All experiments in shake flasks and serum bottles as well as all bioreactor cultivations were carried out in chemically defined medium adapted from Riesenberg et al. [32], containing 13.3 g l^−1^ KH_2_PO_4_, 4.0 g l^−1^ (NH_4_)_2_HPO_4_, 1.7 g l^−1^ citric acid (autoclaved), 1.2 g l^−1^ MgSO_4_ * 7 H_2_O, 0.10 g l^−1^ Fe(III)citrate, 0.0084 g l^−1^ EDTA, 0.013 g l^−1^ Zn(CH_3_COO)_2_ * 2 H_2_O, 0.0025 g l^−1^ CoCl_2_ * 6 H_2_O, 0.015 g l-^1^ MnCl_2_ * 4 H_2_O, 0.0012 g l^−1^ CuCl_2_ * 2 H_2_O, 0.0030 g l^−1^ H_3_BO_3_ and 0.0025 g l^−1^ Na_2_MoO_4_ * 2 H_2_O (sterile filtered). The carbon source was added from a 10 × concentrated stock. Glucose was used at 8 g l^−1^ in isobutanol adaptation experiments, at 20 g l^−1^ in the strain screening experiments and at 50 g l^−1^ in batches and fed-batches. An initial lactose concentration of 50 g l^−1^ was used in pulsed fed-batches.

The feed medium contained 800 g l^−1^ glucose and MgSO_4_ * 7 H_2_O (5.0 g l^−1^), Fe(III)citrate (0.42 g l^−1^), EDTA (35 mg l^−1^), Zn(CH_3_COO)_2_ * 2 H_2_O (54.0 mg l^−1^), CoCl_2_ * 6 H_2_O (11 mg l^−1^), MnCl_2_ * 4 H_2_O (63 mg l^−1^), CuCl_2_ * 2 H_2_O (5.0 mg l^−1^), H_3_BO_3_ (13 mg l^−1^), Na_2_MoO_4_ * 2 H_2_O (11 mg l^−1^) or 250 g l^−1^ lactose and MgSO_4_ * 7 H_2_O (1.6 g l^−1^), Fe(III)citrate (0.13 g l^−1^), EDTA (11 mg l^−1^), Zn(CH_3_COO)_2_ * 2 H_2_O (17.0 mg l^−1^), CoCl_2_ * 6 H_2_O (3.3 mg l^−1^), MnCl_2_ * 4 H_2_O (20 mg l^−1^), CuCl_2_ * 2 H_2_O (1.6 mg l^−1^), H_3_BO_3_ (3.9 mg l^−1^) and Na_2_MoO_4_ * 2 H_2_O (3.3 mg l^−1^). Feed medium was pulsed to the cultures upon substrate depletion to restore a concentration of 50 g l^−1^.

Liquid and solid media were supplemented with 50 µg ml^−1^ kanamycin or 100 µg ml^−1^ ampicillin as necessary.

Glycerol stocks for the storage at − 80 °C of all strains were prepared by mixing 700 µl of liquid overnight culture with 300 µl sterile glycerol (75%).

### Preparation of medium containing sour whey

Spray-dried sour whey powder was kindly provided by NÖM AG (Niederösterreichische Molkerei AG, Baden, Austria). For the batch medium, 67.5 g sour whey powder was dissolved in 1 l distilled water and heated to 70 °C for 20 min. After cooling down, 13.3 g l^−1^ KH_2_PO_4_, 4.0 g l^−1^ (NH_4_)_2_HPO_4_ and 1.7 g l^−1^ citric acid were added, and the pH was adjusted to 6.8. The medium was centrifuged at 14 000 rpm and 21 °C for 10 min, sterile filtered and supplemented with trace elements at the same concentration as the defined medium.

The whey feed was prepared by dissolving 337.5 g sour whey per 1 l dissolved water, followed by heating, centrifugation and sterile filtration. MgSO_4_ * 7 H_2_O (1.6 g l^−1^), Fe(III)citrate (0.13 g l^−1^), EDTA (11 mg l^−1^), Zn(CH_3_COO)_2_ * 2 H_2_O (17.0 mg l^−1^), CoCl_2_ * 6 H_2_O (3.3 mg l^−1^), MnCl_2_ * 4 H_2_O (20 mg l^−1^), CuCl_2_ * 2 H_2_O (1.6 mg l^−1^), H_3_BO_3_ (3.9 mg l^−1^) and Na_2_MoO_4_ * 2 H_2_O (3.3 mg l^−1^) were added as for the defined feed media [14].

### Construction of plasmids and strains

For all cloning steps in this study GoldenMOCS, a Golden Gate-based cloning system, was used [33, 34] and all primers and gBlocks were purchased from Integrated DNA Technnologies (IA, USA).

The genes *alsS* from *Bacillus subtilis* and *budB* from *Enterobacter cloacae subsp. dissolvens* DSM 16,657 were amplified as described elsewhere [14]. The genes *kdcA*, *adhA* and *adhA_mut* from *Lactobacillus lactis* and *ilvD* and *ilvC_mut* from *E. coli* W were purchased as gBlocks from IDT (IA, USA). The genes were flanked with fusion site 2 (FS2) and fusion site 3 (FS3). A colony PCR with Q5 High-Fidelity DNA Polymerase (New England Biolabs, MA, USA) was used to amplify *ilvC* from *E. coli* W and fusion sites 2 and 3 were added with the primers.

The PCR fragments and gBlocks were used for individual BB1 (backbone 1) assemblies [34]. The correct plasmid assembly was verified by restriction digest and Sanger sequencing (Microsynth AG, Switzerland) using the primers seq_fw and seq_rev and additional primers as indicated (Supplementary Material, Table S1).

**Fig. 2:**
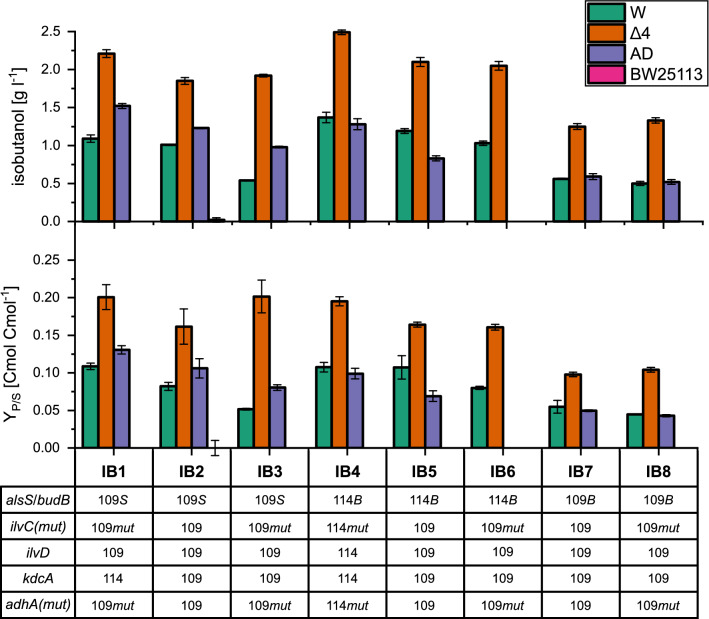
Results of strain and construct screening for isobutanol production in *Escherichia coli* BW25113, *E. coli* W (W), *E. coli* W *∆ldhA ∆adhE ∆pta ∆frdA* (Δ4) and *E. coli* W adapted to high isobutanol concentrations (AD) on minimal medium with 20 g l^−1^ glucose. Results are given as means and standard deviations of biological triplicates. The indication of overexpressed genes are as follows: *alsS* (S) from *Bacillus subtilis* or *budB* (B) from *Enterobacter cloacae* subsp*. dissolvens* are acetolactate synthases, *ilvC* from *E. coli* W serves as ketol-acid reductoisomerase, *ilvC_mut* (mut) indicates a mutated form using NADH rather than NADPH as a cofactor, *ilvD* is dihydroxy-acid dehydratase from *E. coli* W, *kdcA* from *Lactococcus lactis* is α-ketoisovalerate decarboxylase and *adhA* from *L. lactis* is the alcohol dehydrogenase with the mutated form *adhA_mut* (mut) that displays higher catalytic activity. Constitutive promotors of the Anderson constitutive promotor library are indicated by 109 (J23109, weaker promotor) and 114 (J23114, stronger promotor). AD IB6 was not positively transformed and thus not tested

Subsequently, each gene was assembled in BB2 with a constitutive promotor from the Anderson constitutive promotor library (J23109 or J23114) [35] and a synthetic terminator (B1001). These individual expression cassettes were finally used for BB3 assembly resulting in plasmids containing the full pathway consisting of five genes on one plasmid. Different promotor and gene combinations were used to construct a library of eight different vectors (Fig. 2). After BB2 and BB3 assembly, restriction digest was performed to verify for correct integration.

### Adaptation to higher isobutanol concentrations

To enable *E. coli* W to grow in the presence of higher isobutanol concentrations, the strain was adapted by cultivation on increasing isobutanol concentrations. The initial isobutanol concentration was 5 g l^−1^ and was increased in steps of 1 g l^−1^ up to 10 g l^−1^. Then, isobutanol concentrations of 12 g l^−1^ and from 15 g l^−1^ to 23 g l^−1^ steps of 2 g l^−1^ were applied. As soon as growth was observed for a certain condition, the cells were transferred to a higher concentration and glycerol stocks were prepared. The cells were grown at 37 °C and 200 rpm in 100 ml Erlenmeyer flasks with 20 ml defined medium containing 8 g l^−1^ glucose.

### Preparation of precultures

All strains and constructs were stored at − 80 °C in 23% glycerol. For cultivations, they were streaked onto LB agar plates containing 50 µg ml^−1^ kanamycin and incubated overnight at 37 °C. A single colony was used for inoculation of 500 ml shake flasks containing 50 ml of LB medium or 2xLB medium for serum bottles or bioreactor cultivation, respectively. The preculture was incubated overnight at 37 °C and 230 rpm. The cells were centrifuged at 4800 rpm for 10 min at room temperature and washed with 25 ml of sterile 0.9% (w/v) NaCl. After resuspension in 5 ml 0.9% (w/v) NaCl, the optical density at 600 nm (OD_600_) was measured and the appropriate volume of preculture to reach an initial OD_600_ of 1 was transferred to the bioreactor. The same procedure was used for shake flask and serum bottle experiments, but the initial OD_600_ was 0.5.

### Strain and construct screening

For exact isobutanol quantification, the construct screening was carried out in 120 ml serum bottles sealed with butyl rubber septa to avoid loss by evaporation. The bottles were filled with 20 ml defined medium with a glucose concentration of 20 g l^−1^. The bottles were incubated at 37 °C and 180 rpm. Samples were taken after 24 h and 48 h for OD_600_ and HPLC measurements.

### Cultivations in bioreactors

Bioreactor cultivations were performed in duplicate in a DASbox^®^ Mini Bioreactor system (Eppendorf AG, Hamburg, Germany). The working volume was 200 ml and all cultivations were carried out at 30 °C. The pH was maintained at 6.8 by addition of 12.5% (v/v) NH_4_OH with a MP8 Multipumpmodule (Eppendorf AG, Hamburg, Germany) and monitored by a pH electrode EasyFerm Plus K8 120 (Hamilton, Reno, NV, USA). The concentration of dissolved oxygen was monitored by a VisiFerm DO 120 probe (Hamilton, Reno, NV, USA). The agitator speed was kept constantly at 500 or 800 rpm for microaerobic cultivations of *E. coli* W Δ*ldhA* Δ*adhE* Δ*pta* Δ*frdA* (Δ4) IB4 and *E. coli* W (W) IB4, respectively, and adapted from 800 to 2000 rpm in aerobic cultivations. The gassing rate was set to 0.2 vvm (2.4 sl h^−1^) to avoid isobutanol stripping in batches. During aerobic cultivations, air was mixed with oxygen to maintain a dissolved oxygen concentration above 30%. For isobutanol stripping in pulsed fed-batches, the gassing rate was increased to 1 vvm (12 sl h^−1^) after the first batch. To collect isobutanol from the reactor off-gas, the gas stream was flushed through cooled wash bottles on ice containing 500 ml distilled water and 5 g l^−1^ citric acid. For calculation of the absolute isobutanol concentrations, amounts in the reactor were added to the amounts in the wash bottles. Off-gas analysis for O_2_ and CO_2_ was carried out using the gas analyzer module GA4 (Eppendorf AG, Hamburg, Germany).

Samples of 4 ml were taken regularly and the optical density at 600 nm was measured to estimate biomass growth. The samples were centrifuged at 14 000 rpm for 5 min and the supernatant was used for HPLC analysis of substrate and product concentrations.

### Determination of biomass

Cell dry weight (CDW) was determined gravimetrically in duplicates from bioreactor samples at the end of the batch phases. To this end, 4 ml of culture broth was centrifuged at 4800 rpm and 4 °C for 10 min, washed with 4 ml deionized water and centrifuged again. The pellet was dried in pre-weighed glass tubes for at least 72 h at 105 °C. The optical density at 600 nm (OD_600_) was measured in a spectrophotometer (Genesys™ 20, Thermo Scientific, Waltham, Massachusetts, USA) against a water blank. The correlation between OD_600_ and cell dry weight was used to estimate the cell concentration for all time points except end of batch and feed phases.

### HPLC analysis

Sugars, organic acids, and alcohols were determined using an Aminex HPX-87H column (300 × 7.8 mm, Bio-Rad, Hercules/CA, USA) in an Ultimate 3000 system (Thermo Scientific, Waltham/MA, USA). 4 mM H_2_SO_4_ was used as a mobile phase at 60 °C and a flow of 0.6 ml min^−1^ for 40 min and the injection volume was 10 µl. Metabolites were detected using a refractive index (Refractomax 520, Thermo Scientific, Waltham/MA, USA) and a DAD detector (Ultimate 3000, Thermo Scientific, Waltham/MA, USA). Chromeleon 7.2.6 Chromatography Data System (Thermo Scientific, Waltham/MA, USA) was used for control, monitoring and evaluation of the analysis.

For sample preparation, 450 µl of cell-free culture supernatant was mixed with 50 µl of 40 mM H_2_SO_4_ and centrifuged for 5 min at 14 000 rpm at 4 °C. The supernatant was used for analysis and standards were treated the same way. A 5-point calibration was used for substrate and metabolite concentrations in the samples.

## Results

### Strain construction and screening

The goal of this study was to establish an *E. coli* system for isobutanol production in chemically defined medium and alternative raw materials such as cheese whey. To that end, we created a construct library expressing each gene individually under a constitutive promotor. Additionally, different strain backgrounds were tested to find the best construct–strain combination for efficient isobutanol production.

For the assembly of the isobutanol production pathway (Fig. 1), acetolactate-synthase, ketol-acid reductoisomerase, dihydroxy-acid dehydratase, α-ketoisovalerate decarboxylase and alcohol dehydrogenase were constitutively overexpressed using promotors of different strength from the Anderson constitutive promotor library [35]. This enabled the expression fine-tuning of each gene in an independent expression cassette. Two different types of constitutive promotors were used, the medium strength BBa_J23114 (114p) and the weaker promotor BBa_J23109 (109p). Plasmid assemblies were found to be challenging, as some promotor–gene combinations did not yield positive clones indicating the burden posed to the cell by expression of this pathway. A library containing eight different genetic constructs was created.

Investigating the influence of the strain background on isobutanol production, construct IB2 was tested in two different strains: *E. coli* W and K12-BW25113. To avoid evaporation and for the exact determination of product concentrations, serum bottles were used.

*E. coli* W showed growth and isobutanol production on chemically defined medium containing 20 g l^−1^ glucose (Fig. 2). In contrast, no isobutanol was produced in *E. coli* K12-BW25113. This strain showed growth, but glucose utilization was low and high amounts of acids were secreted (Supplementary Material, Table S2).

Subsequently, the influence of isobutanol pathway gene expression was studied. For an improved strain background, all eight constructs were screened in three *E. coli* W-derived strains: the parental *E. coli* W, *E. coli* W Δ*ldhA* Δ*adhE* Δ*pta* Δ*frdA* (Δ4) and *E. coli* W adapted to high isobutanol concentrations (AD). The deletion of by-product formation pathways in Δ4 should increase the driving force towards product formation and isobutanol cytotoxicity should be overcome using the adapted strain. After 30 sequential transfers to increased isobutanol concentrations, the adapted strain was able to grow in the presence of 21 g l^−1^ isobutanol (Supplementary Material, Table S3). *E. coli* W AD showed improved growth at higher isobutanol concentrations than the parental strain and this effect was more propagated at lower temperatures of 30 °C (data not shown). The screening showed that isobutanol was produced regardless of the construct composition, indicating the suitability of the constitutive expression system. Because genes were expressed as individual cassettes, we could study the influence of different promotors and genes on isobutanol production (Fig. 2). For instance, the expression of *alsS* rather than *budB* as acetolactate synthase (IB3 vs. IB8) led to higher product concentrations and yields. A stronger promotor for *budB* increased the isobutanol yield (IB5 versus IB7 and IB6 versus IB8) to the level reached with weaker expression of *alsS* (IB6 versus IB3). Apart from the acetolactate synthase, constructs using either the wild-type or mutated versions of *ilvC* and *adhA* showed similar isobutanol yields (IB5 versus IB6 and IB7 versus IB8). Additionally, an increased promotor strength of *kdcA* led to similar or increased isobutanol yields compared to a weaker promotor in *E. coli* W Δ4 and *E. coli* W, respectively (IB1 versus IB3).

The knockout of the mixed acid fermentation pathways (Δ4) resulted in decreased by-product formation, which in turn led to an increase of the isobutanol production and yield for all constructs (Supplementary Material, Table S2). For most constructs, there was no difference in the product yield between the *E. coli* W strain which was adapted to high isobutanol concentrations (AD) and the W strain. However, in the construct IB5, the obtained isobutanol yield was even 36% lower in the AD strain compared to the W strain.

For the selection of a suitable production strain, total isobutanol production and yield were evaluated (Fig. 2). The highest yield in combination with the highest titer and highest glucose uptake was achieved in *E. coli* W Δ4 IB4 expressing all genes from the medium strong 114p promotor and carrying *budB* as acetolactate synthase. Additionally, both *ilvC* and *adhA* were present in the mutated forms and utilized NADH as a cofactor.

### Isobutanol production in aerobic and microaerobic batch cultivations

Microorganisms typically produce alcohols in the absence of oxygen under anaerobic or microaerobic conditions. In the strain screening experiments, microaerobic conditions in sealed serum bottles were successfully used for isobutanol production. Anaerobic conditions led to a growth defect in *E. coli* W Δ4 and accumulation of high amounts of acids in *E. coli* W. To investigate the effect of oxygen availability on isobutanol production under controlled conditions, *E. coli* W IB4 and Δ4 IB4 were tested in batch experiments. Based on the initial construct screening, microaerobic conditions (dissolved oxygen concentration of 0% in exponential phase) were compared to aerobic conditions. For all bioreactor cultivations, isobutanol stripping was monitored by a retention system.

**Fig. 3:**
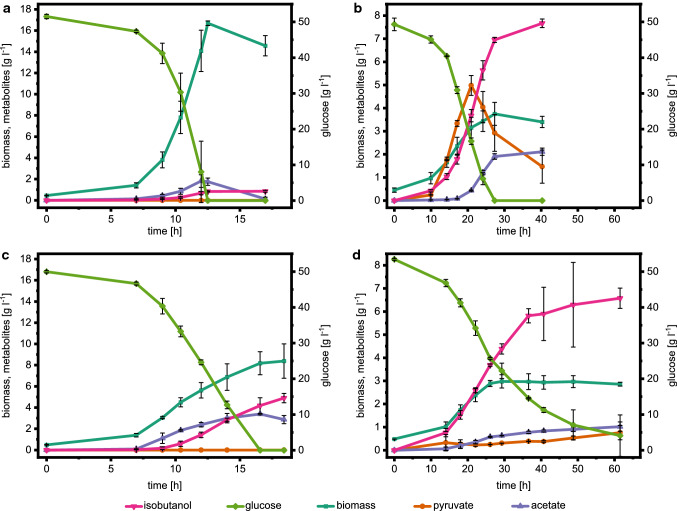
Substrate and metabolite concentrations in batch experiments on defined medium with 50 g l^−1^ glucose of **a** *E. coli* W IB4 under aerobic conditions, **b** *E. coli* W *∆ldhA ∆adhE ∆pta ∆frdA* IB4 under aerobic conditions, **c** *E. coli* W IB4 under microaerobic conditions and **d** *E. coli* W *∆ldhA ∆adhE ∆pta ∆frdA* IB4 under microaerobic conditions. Means of biological duplicates are shown and error bars represent standard deviations

**Fig. 4:**
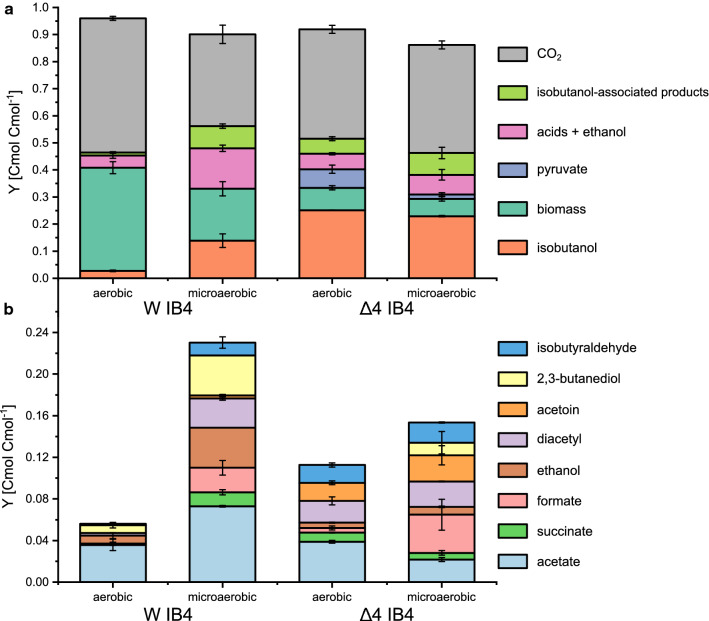
Product yields [Cmol product Cmol^−1^ glucose] in batch experiments of *E. coli* W IB4 (left) and *E. coli* W *∆ldhA ∆adhE ∆pta ∆frdA* IB4 (right) under aerobic and microaerobic conditions in minimal medium with 50 g l^−1^ glucose. In **a**, isobutyraldehyde, 2,3-butanediol, acetoin and diacetyl are summarized as isobutanol-associated products and acetate, formate, succinate and ethanol are summarized as acids + ethanol. Detailed by-product yields are shown in **b**. Acetol (hydroxyacetone) and 1,2-propanediol were also analyzed but not detected. Means of biological duplicates are shown and error bars represent standard deviations

Figure 3 shows the comparison of isobutanol production under aerobic and microaerobic conditions in *E. coli* W and Δ4. Under aerobic conditions, *E. coli* W mainly produced biomass and CO_2_ and only minor amounts of isobutanol (0.9 ± 0.1 g l^−1^), but under microaerobic conditions isobutanol production increased to 4.9 ± 0.4 g l^−1^. In contrast, the aerobic and microaerobic cultures of *E. coli* W Δ4 achieved significantly higher isobutanol titers of 7.7 ± 0.2 g l^−1^ and 6.6 ± 0.4 g l^−1^, respectively. Moreover, deletion of mixed acid fermentation pathways resulted in a decreased biomass yield in *E. coli* W Δ4 (Fig. 4a), as shown before [14]. Nevertheless, the specific glucose uptake and isobutanol production rate of *E. coli* W Δ4 was significantly higher compared to *E. coli* W (Table 2). Additionally, *E. coli* W Δ4 produced significantly less by-products under all conditions compared to *E. coli* W and the isobutanol yield increased by 80% to 0.25 Cmol Cmol^−1^. However, all by-products combined still accounted for 12% of the total carbon in aerobic cultures of *E. coli* W Δ4 (Fig. 4b). Despite deletion of phosphate acetyl transferase (*pta*), acetate was still a major by-product during aerobic cultivation of this strain. Additionally, isobutyraldehyde, diacetyl, 2,3-butanediol and acetoin associated with the isobutanol production pathway were detected as unspecific by-products. Interestingly, also pyruvate accumulated in significant amounts (5.0 ± 0.4 g l^−1^) during aerobic cultivation of *E. coli* W Δ4, while the wild-type strain did not secrete pyruvate.

**Table 2:** Maximum volumetric (*r*_Iso_) and specific (*q*_Iso_) isobutanol production and maximum volumetric (*r*_S_) and specific (*q*_S_) glucose uptake rates of *E. coli* W IB4 (W) and *E. coli* W *ΔldhA ΔadhE Δpta ΔfrdA* IB4 (Δ4) in batch experiments on chemically defined medium with 50 gl^−1^ glucose

Strain	Condition	*r*_Iso_ g l^−1^ h^−1^)	*q*_Iso_ (g g^−1^ h^−1^)	*r*_S_ (g l^−1^ h^−1^)	*q*_S_ (g g^−1^ h^−1^)	OUR (mmol l^−1^ h^−1^)	*q*_O2_ (mmol g^−1^ h^−1^)
W	Aerobic	0.33 ± 0.04	0.02 ± 0.00	15.7 ± 0.3	1.29 ± 0.01	263 ± 40	17.1 ± 1.6
	Microaerobic	0.75 ± 0.01	0.11 ± 0.01	6.1 ± 0.6	1.50 ± 0.22	49.9 ± 21	9.5 ± 1.2
Δ4	Aerobic	0.62 ± 0.04	0.19 ± 0.01	3.7 ± 0.1	1.70 ± 0.08	21.3 ± 0.4	8.7 ± 2.5
	Microaerobic	0.27 ± 0.04	0.15 ± 0.01	2.1 ± 0.4	1.01 ± 0.04	9.8 ± 0.8	3.9 ± 1.3

For aerobic conditions, dissolved oxygen was maintained above 30%. Microaerobic conditions were maintained at constant stirrer speed of 800 rpm (W) and 500 rpm (Δ4) and dissolved oxygen dropped to 0% in the exponential phase. Mean values and standard deviations were calculated from biological duplicates

### Isobutanol production in pulsed fed-batch cultivations

Upon successful production of isobutanol in batch experiments, we aimed to further increase product titers using fed-batch cultivations. Initially, we sought to establish a fed-batch cultivation with a linear feeding profile. However, a stable process could not be achieved and high variations in isobutanol and biomass concentration were observed (data not shown). Therefore, we performed pulsed fed-batches for process intensification, which have been successfully used for microbial production of platform chemicals such as 2,3-butanediol [14]. Aerobic conditions were selected for the pulsed fed-batches as initial batch cultivations had yielded the highest isobutanol titers and yields for *E. coli* W Δ4. All subsequent pulsed fed-batches were carried out with this strain under aerobic conditions. In the first batch phase, low gassing rates were applied. To prevent cell death caused by isobutanol toxicity in the subsequent phases, the volatile compound was stripped by the increase of gassing rates (Supplementary Material, Tables S5, S6 and S7).

**Fig. 5:**
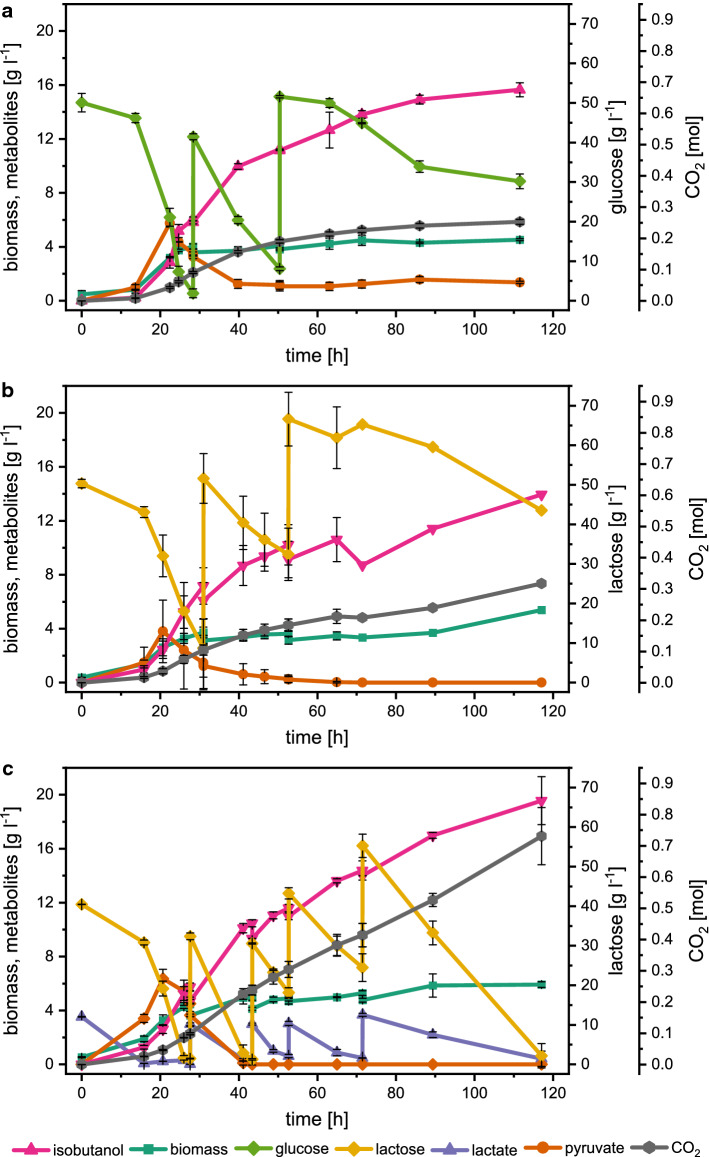
Pulsed fed-batches of *E. coli* W *∆ldhA ∆adhE ∆pta ∆frdA* IB4 under aerobic conditions on **a** minimal medium with 50 g l^−1^ glucose, **b** minimal medium with 50 g l^−1^ lactose and **c** cheese whey medium. Substrate uptake and metabolite as well as CO_2_ formation are shown. Upon depletion of the carbon source, new medium was pulsed to obtain substrate concentrations of 50 g l^−1^. Means of biological duplicates are shown and error bars represent standard deviations

Glucose pulses resulted in the production of 15.6 ± 0.5 g l^−1^ isobutanol (Fig. 5a). In the later phases (batch 2 and 3), the carbon flux shifted from biomass to isobutanol production and formation of CO_2_ (Supplementary Material, Table S5). Batch 2 showed the highest isobutanol production rate (0.25 g l^−1^ h^−1^), whereas batch 3 showed the highest isobutanol yield (52% of the theoretical maximum). In batch 3, glucose uptake and isobutanol production rates decreased. (Supplementary Material, Table S5).

With the final production of 15.6 g l^−1^ isobutanol, the process mode of a pulsed fed-batch was found to be suitable for cheap, reproducible, and easily feasible production.

### Production of isobutanol from lactose and cheese whey

The production cost for fuel alcohols could further be decreased using alternative raw materials. Therefore, we investigated whether spray-dried sour whey is a suitable substrate for isobutanol production.

For comparison of the process performance, a reference process was carried out on defined medium with lactose as carbon source (Fig. 5b). Using pure lactose instead of glucose as carbon source decreased the final isobutanol titer by 10% (Table 3). In contrast, the fed-batch using cheese whey showed a higher isobutanol titer of 19.6 ± 1.8 g l^−1^ representing an increase of 26 and 40% compared to defined medium with glucose and lactose, respectively (Table 3). In contrast to synthetic media, constant substrate uptake and isobutanol production are ensured over a longer time period, resulting in an overall higher isobutanol productivity (Table 3). The isobutanol yield reached 0.26 Cmol Cmol^−1^, which is 39% of the theoretical maximum. In contrast to the defined medium, lactose utilization was accelerated in the cheese whey process. In the second batch of the whey process, lactose uptake peaked at 1.97 g l^−1^ h^−1^, a twofold increase compared to the defined lactose medium (Supplementary Material, Tables S6 and S7). Moreover, lactose and lactate co-utilization was observed in whey based medium (Fig. 5c). Lactate co-utilization can increase NADH availability, which is beneficial for isobutanol production. The availability of additional substrates in cheese whey resulted in a slightly increased biomass yield compared to synthetic medium (Supplementary Material, Tables S5, S6 and S8).

**Table 3:** Mean total volumetric (*r*_Iso_) and specific (*q*_Iso_) isobutanol production, volumetric (*r*_S_) and specific (*q*_S_) glucose uptake rates, isobutanol (*Y*_Iso/S_) biomass (*Y*_X/S_) and CO_2_ (*Y*_CO2/S_) yield and total carbon recoveries of *E. coli* W *∆ldhA ∆adhE ∆pta ∆frdA* IB4 in glucose, lactose and cheese whey pulsed fed-batch experiments

	Glucose	Lactose	Cheese whey
Isobutanol (g l^−1^)	15.6 ± 0.5	14.0	19.6 ± 1.8
*r*_Iso_ (g l^−1^ h^−1^)	0.14 ± 0.01	0.12	0.17 ± 0.02
*q*_Iso_ (g g^−1^ h^−1^)	0.04 ± 0.01	0.04	0.04 ± 0.01
*r*_S_ (g l^−1^ h^−1^)	0.92 ± 0.03	0.70	1.32 ± 0.02
*q*_S_ (g g^−1^ h^−1^)	0.27 ± 0.01	0.24	0.31 ± 0.01
*Y*_Iso/S_ (Cmol Cmol^−1^)	0.25 ± 0.02	0.30	0.26 ± 0.04 0.24 ± 0.04*
*Y*_X/S_ (Cmol Cmol^−1^)	0.045 ± 0.002	0.073	0.063 ± 0.009 0.057 ± 0.008*
*Y*_CO2/S_ (Cmol Cmol^−1^)	0.56 ± 0.05	0.58	0.53 ± 0.05 0.48 ± 0.04*
C recovery (%)	102 ± 6	102	90 ± 1.5

Mean values and standard deviations were calculated from duplicate experiments. As one lactose cultivation failed after batch 2, the parameters are calculated from one replicate. For the cheese whey process, yields were calculated considering lactose or the sum of lactose and lactate (*) as substrate

Pyruvate accumulated in the first batch and was subsequently consumed in the subsequent process phases. Cultures using lactose as carbon source completely consumed pyruvate, whereas a portion of pyruvate remained in the culture broth when glucose was the substrate. Since pyruvate accumulation is generally associated with metabolic stress [36], it is possible that higher specific substrate uptake rates for glucose increased metabolic burden compared to lactose cultures (Supplementary Material, Tables S5 and S7). Comparable to batch experiments, the by-products acetate, succinate, diacetyl, acetoin and isobutyraldehyde were detected in different amounts in the culture broth (Supplementary Material, Table S4).

## Discussion

The comparison of two different strain backgrounds revealed the suitability of *E. coli* W strains for isobutanol production using chemically defined medium. The superior performance of this strain can probably be attributed to a high stress tolerance and fast growth [39]. *E. coli* K12-BW25113 has been reported as an efficient isobutanol producer on complex media [5, 6, 37], but isobutanol production on defined medium failed in this study. This discrepancy in the performance of a strain on defined and complex medium suggests that the right screening platform (i.e., chemically defined medium) should be chosen for further strain and process development [14].

Especially on minimal medium, a balance between enzyme expression and cell fitness has to be established for microbial production of platform chemicals [14]. Pathway construction as individual cassettes without inducible promotors was suitable for isobutanol production and revealed the influence of single gene expression levels. For the first enzyme in the pathway, acetolactate synthase, the tenfold higher activity of AlsS compared to BudB [40] probably accounted for increased isobutanol yields. We aimed to further increase acetolactate expression by using *alsS* with a stronger promotor, but this assembly was technically not feasible due to metabolic burden by enhanced enzyme expression. Stronger expression of *alsS* might not necessarily lead to improved isobutanol production, since a construct expressing *budB* rather than *alsS* from the same promotor was found to yield higher 2,3-butanediol production [14]. These results indicate that keeping a balance between strain fitness and product formation is especially important on defined medium. The expression of *kdcA* and *adhA* is a potential bottleneck, since the intermediate product isobutyraldehyde is both very toxic and volatile [41]. That the stronger expression of *kdcA* improved isobutanol production suggests that the high affinity towards isobutyraldehyde in the subsequent enzyme AdhA_mut [18] allowed for efficient conversion of the toxic intermediate in the best producing strain. Considering product yield and isobutanol titer, IB4 with medium strong expression levels for all genes was found to be the most suitable construct.

In accordance with other studies, adaptation of *E. coli* W to isobutanol did increase the tolerance concentration up to which growth was possible significantly. However, the production characteristics were not improved in the adapted strain [3, 42, 43]. In contrast, the toxicity of isobutanol led to the inhibition of biomass formation in the non-adapted strain, which was one key factor to enable efficient isobutanol production. Similarly, increasing the driving force of pyruvate availability by deletion of competing pathways led to higher alcohol production [5, 44–46]. Therefore, the combination of inhibited growth by toxic isobutanol production and the availability of pyruvate were crucial for efficient isobutanol production, while adaptation of *E. coli* did not improve product formation.

The driving force can also be improved by increased NADH availability. Since this availability is directly related to oxygen supply in a cell, different aeration strategies can greatly influence product formation [44, 47]. In *E. coli* W, isobutanol production could be improved by microaerobic cultivation, whereas the reduction of oxygen supply led to decreased substrate utilization and production rates in *E. coli* W Δ4. Considering microbial production of other alcohols, the reduction of oxygen availability was successful for 2,3-butanediol and n-butanol production [14, 15, 44], whereas isobutanol and isopropanol could be produced under aerobic conditions [6, 48].

That the deletion of mixed acid fermentation pathways in *E. coli* W Δ4 increased aerobic isobutanol production is somewhat surprising, as these pathways are usually repressed under aerobic conditions. In this scenario, pyruvate accumulates, which indicates metabolic stress [36] and is associated with isobutanol production. Isobutanol toxicity is, among other factors, based on quinone inhibition, which activates the aerobic respiration control protein ArcA [36]. By repressing aerobic enzymes such as pyruvate dehydrogenase, ArcA activation leads to pyruvate accumulation [49] and increased NADH/NAD^+^ ratios [36]. Additionally, deletions of *ldhA* and *pta* have been shown to increase pyruvate formation [50, 51]. In other words, the combination of increased NADH availability, reduced by-product formation and higher pyruvate availability led to increased isobutanol titers and yields under aerobic conditions in *E. coli* W Δ4 [3, 36]. Since the described toxicity mechanism is unique for higher alcohols, the effect of aerobic production is not directly transferable to other alcohols.

In accordance with our findings, reduced acetate accumulation by deletion of *pta* was previously shown and an additional knockout of pyruvate oxidase *poxB* was not reported to increase product formation [6]. The formation of other by-products is probably a result of lacking specificity of the individual isobutanol pathway enzymes or utilization of pathway intermediates as substrates by native *E. coli* enzymes [52, 53] (Fig. 1). In detail, diacetyl is produced by spontaneous decarboxylation of acetolactate and is converted to acetoin and 2,3-butanediol [54] by native or overexpressed enzymes with indistinct substrate patterns (Fig. 1). Requiring NADH as a cofactor, the formation of 2,3-butanediol from acetoin is one observation that reflects the redox status of the cell. Similarly, NADH availability influences the conversion of isobutyraldehyde to isobutanol. A low activity of AdhA might have caused isobutyraldehyde accumulation, but AdhA_mut was reported to have a high affinity toward its substrate [18]. In accordance, the toxic intermediate was mainly found in the wash bottles, which suggests that its high volatility caused stripping from the culture.

Aerobic cultivation of *E. coli* W Δ4 yielded the highest isobutanol of 38% of the theoretical maximum which is comparable to approximately 36% previously reported for defined medium [5]. Systems relying on complex media components using yeast extract concentrations of up to 25 g l^−1^ showed higher yields [5, 6, 18]. Moreover, the addition of yeast extract has been shown to enhance isobutanol productivity (2.8-fold increase, Table 1) [6]. For comparable defined production systems, data are only available for non-toxic diol production. Using the same strain background (*E. coli* W Δ4) for 2,3-butanediol production, a fivefold higher production rate was reported [14]. That 76% of the theoretical yield was reached suggests limited production due to isobutanol toxicity in this study.

The availability of additional nutrients in cheese whey resulted in an increased final product titer, which is beneficial for further cost-effective downstream processing [55]. The production of 19.6 g l^−1^ isobutanol is the highest titer obtained on alternative raw materials. Isobutanol yield reached 39% of the theoretical maximum, which is a 1.5- to 2.8-fold increase compared to lignocellulosic hydrolysates [25, 26]. These promising results were obtained by keeping cells at high performance for an extended time period and thereby increasing overall productivity. We speculated that a strategy combining different factors is key for successful isobutanol production. One factor might be the optimum concentration of isobutanol in the fermentation broth to favor product over biomass formation due to isobutanol toxicity. By applying pulses rather than a constant feeding profile, the process is operated at the maximal possible uptake and production rate, which might also improve product formation. Similarly, high glucose concentrations at the beginning of every pulse could also slightly inhibit cell growth and favor isobutanol production. A comparison of state-of-the-art processes for isobutanol production is shown in Table 1.

In this work, investigation of isobutanol production on defined medium and cheese whey as an alternative carbon source provided valuable information for further investigation on the way to potential industrial applications. In Table 4, we calculated commercial indicators for different production scenarios from this study and literature reports [6]. Additionally, we estimated the production cost if yeast extract is replaced by an alternative nutrient source. To this end, the amounts of utilized carbon source and media additives were calculated based on reported yields and product titers. These amounts were used to estimate the media cost [56] and the minimum price at which isobutanol has to be sold to cover these costs. Since cheese whey and corn steep liquor are waste products, these media were assumed not to generate costs. On the contrary, the costs for safe disposal of whey are difficult to estimate [30, 58], but can range from 0.6 to 4.4 (US) cent per pound of cheese processed [59]. Costs for media components, bioreactor operation and downstream processing have not been considered, as they do not depend on the substrate utilized for isobutanol production and would therefore add similar but hard to estimate costs to all scenarios.

**Table 4:** Comparison of estimated media cost and minimal selling price for microbial isobutanol production

Medium	C-Source	Complex media additive	Isobutanol titer (g l-^1^)	Isobutanol yield (g g^−1^)	Media cost ($/m^3^)	Minimum isobutanol selling price ($/kg)	References
Defined	103 g l^−1^ glucose	–	16	0.15	48.6	3.1	This study
Cheese whey	254 g l^−1^ spray-dried cheese whey	–	20	0.08	0.00	0.0	This study
Complex	176 g l^−1^ glucose	25 g l^−1^ yeast extract	51	0.29	1644	32	[6]
Alternative complex	176 g l^−1^ glucose	250 g l^−1^ corn steep liquor	51	0.29	83	1.7	Theoretical

Prices for glucose and yeast extract were obtained from Rodrigues et al. (2007) and converted to US$ at the current exchange rate of 1.18 US$ per € [56]. Corn steep liquor and cheese whey were assumed to cost 0.00 $ since they are waste products. The alternative complex medium is based on yields reported by Baez et al. [6], but yeast extract was assumed to be replaced by corn steep liquor as successfully shown by Saha (2006) [57]. The calculation of the minimum selling price is greatly simplified, since only the main media components were used for calculation. Additional costs such as for energy, downstream processing or other media additives were not considered

Table 4 shows that the addition of yeast extract greatly influences the total media cost. Its replacement by other raw materials such as corn steep liquor (CSL) might be a promising alternative. However, Saha (2006) reported that CSL had to be used at a concentration of 50 g l^−1^ to achieve a similar effect as with 5 g l^−1^ yeast extract [57]. The high concentrations that need to be applied could limit the use of this media additive. Alternatively, yeast extract could also be purchased at lower prices from breweries, where yeast is a main by-product [60, 61]. However, yeast biomass is from fermentation processes is frequently used as an animal feedstock. It seems likely that the availability of yeast biomass for a fermentation process yielding a low-price product such as isobutanol is therefore limited in comparison to the higher price that can be obtained when sold as an animal feedstock. Additionally, variations in yeast extract quality could affect process performance and different brewing processes were shown to influence the nutrient composition [60]. Due to additional costs related to the use of a complex media additive, it is more cost-effective to omit additional media components. We suggest using a cheap raw material (e.g., cheese whey) as a carbon source, thereby avoiding costs for glucose or other sugars that increase the total production cost.

The selling price for isobutanol was reported to be around 1750 $/t in 2015 [62]. Comparing this price to the calculated theoretical selling prices shows that only the use of cheese whey production could result in a cost-competitive process.

Further reduction of fermentation cost can be achieved by the omittance of expensive inducers. This reduction can either be achieved by induction systems that rely on cheaper inducers [26] or by the use of constitutive expression as reported in this study.

Typically, plasmid-based expression requires the use of a cost-intensive selection marker such as kanamycin. Genome integration could therefore be a promising goal for future research.

## Conclusion

In this study, isobutanol was efficiently produced in a chemically defined medium due to the choice of a suitable strain background and expression system. Individual expression of each gene under a constitutive promotor allowed for the selection of a suitable construct. The use of the robust *E. coli* W in combination with strain improvement and the investigation of different aeration strategies were key for the development of an efficient production process. Using cheese whey as an alternative raw material in pulsed fed-batches enabled longer process stability and higher isobutanol titers. In future, investigation of other cheap raw materials and waste streams can contribute to the development of cost-effective processes. In this study, isobutanol production on both chemically defined medium and a residual waste stream was demonstrated, which provides valuable information for further development of industrially relevant isobutanol production processes.

## Electronic supplementary material

Below is the link to the electronic supplementary material.

Supplementary file1 (DOCX 34 kb)

## Data Availability

The datasets used and/or analysed during the current study, if not shown in the text or additional files, are available from the corresponding author on reasonable request.
