# Supplementary material for: Metabolic engineering of Escherichia coli W for isobutanol production on chemically defined medium and cheese whey as alternative raw material
Source: J Ind Microbiol Biotechnol. 2020 Oct 17;47(12):1117–32. doi: 10.1007/s10295-020-02319-y (PMC7728641; doi:10.1007/s10295-020-02319-y)
Supplement: Supplementary file 1 — Supplementary file1 (DOCX 34 kb) [file 10295_2020_2319_MOESM1_ESM.docx]

**Table S1** List of primers for used for PCR amplification or sequencing (seq.)

| **Primer name** | **Sequence** | **Purpose** |
| --- | --- | --- |
| ilvC_in_rev | GGAGATATCGAGGCCAG | PCR |
| ilvC_in_fw | CTGGCCTCGATATCTCC | PCR + Seq |
| ilvC_in2_rev | CGGTTTTGCCTGTCTC | PCR + Seq |
| ilvC_in2_fw | GAGACAGGCAAAACCG | PCR |
| FS2_ilvC_out_fw | GATCGGTCTCACATGGCTAACTACTTCAATACACTG | PCR |
| FS3_ilvC_out_rev | GATCGGTCTCAAAGCTTAACCCGCAACAGC | PCR |
| seq_rev | CGTGGACCGATCATACG | Seq |
| seq_fw | GCAGTCCAGTTACGCTG | Seq |
| ilvD_seqin_fw | GATGCTGATGGCTTCCC | Seq |
| kdcA_seqin_rev | GACAGGCCCAGCTCC | Seq |
| kdcA_seqin_fw | GCCCGCACACTGTTG | Seq |

**Table S2** Isobutanol (Y_P/S_), biomass (Y_X/S_) and by-product (Y_BP/S_) yield in *E. coli* BW25113, *E. coli* W, W *∆ldhA ∆adhE ∆pta ∆frdA* (Δ4) and *E. coli* W adapted to 21 g l^-1^ isobutanol. By-products (BP) were defined as acetate, formate, succinate, lactate and ethanol. Means and standards deviations are calculated from triplicate experiments. AD IB6 was not positively transformed and thus not tested.

| construct | Y_P/S_ [Cmol Cmol^-1^] | | | | Y_X/S_ [Cmol Cmol^-1^] | | | | Y_BP/S_ [Cmol Cmol^-1^] | | | |
| --- | --- | --- | --- | --- | --- | --- | --- | --- | --- | --- | --- | --- |
|  | W | Δ4 | AD | BW25113 | W | Δ4 | AD | BW25113 | W | Δ4 | AD | BW25113 |
| IB1 | 0.11  ± 0.01 | 0.20  ± 0.02 | 0.13  ± 0.01 | - | 0.11  ± 0.01 | 0.09  ± 0.01 | 0.09  ± 0.01 | - | 0.42  ± 0.01 | 0.07  ± 0.01 | 0.28  ± 0.02 | - |
| IB2 | 0.08  ± 0.01 | 0.16  ± 0.02 | 0.11  ± 0.01 | 0.00  ± 0.00 | 0.09  ± 0.01 | 0.09  ± 0.01 | 0.09  ± 0.03 | 0.13  ± 0.01 | 0.33  ± 0.02 | 0.08  ± 0.01 | 0.27  ± 0.03 | 0.86  ± 0.06 |
| IB3 | 0.05  ± 0.01 | 0.20  ± 0.02 | 0.08  ± 0.01 | - | 0.10  ± 0.01 | 0.11  ± 0.01 | 0.09  ± 0.01 | - | 0.43  ± 0.02 | 0.09  ± 0.01 | 0.27  ± 0.01 | - |
| IB4 | 0.11  ± 0.01 | 0.20  ± 0.01 | 0.10  ± 0.01 | - | 0.10  ± 0.01 | 0.10  ± 0.01 | 0.08  ± 0.01 | - | 0.25  ± 0.01 | 0.13  ± 0.01 | 0.29  ± 0.01 | - |
| IB5 | 0.11  ± 0.02 | 0.16  ± 0.01 | 0.07  ± 0.01 | - | 0.11  ± 0.01 | 0.09  ± 0.01 | 0.08  ± 0.01 | - | 0.23  ± 0.04 | 0.08  ± 0.01 | 0.24  ± 0.02 | - |
| IB6 | 0.08  ± 0.01 | 0.16  ± 0.01 | - | - | 0.09  ± 0.02 | 0.09  ± 0.01 | - | - | 0.22  ± 0.01 | 0.08  ± 0.01 | - | - |
| IB7 | 0.05  ± 0.01 | 0.10  ± 0.01 | 0.05  ± 0.01 | - | 0.11  ± 0.05 | 0.09  ± 0.01 | 0.08  ± 0.01 | - | 0.38  ± 0.06 | 0.09  ± 0.01 | 0.30  ± 0.02 | - |
| IB8 | 0.04  ± 0.01 | 0.10  ± 0.01 | 0.04  ± 0.01 | - | 0.09  ± 0.01 | 0.08  ± 0.01 | 0.08  ± 0.02 | - | 0.35  ± 0.01 | 0.08  ± 0.01 | 0.30  ± 0.01 | - |

**Table S3 –** Results from isobutanol adaptation experiments. *E. coli* W was sequentially transferred to medium containing increased initial isobutanol concentrations.

| **Initial isobutanol concentration [g l^-1^]** | 0 | 5 | 9 | 15 | 17 | 19 | 21 |
| --- | --- | --- | --- | --- | --- | --- | --- |
| **Growth after transfer n° (total)** | 1 | 4 | 5 | 6 | 17 | 22 | 30 |

**Table S4** Maximal by-product concentrations of *E. coli* W *∆ldhA ∆adhE ∆pta ∆frdA* IB4 in pulsed fed-batch experiments. Means and standards deviations are calculated from duplicate experiments.

| **By-product** | **Maximum concentration [g l^-1^]** | | |
| --- | --- | --- | --- |
|  | **glucose fed-batch** | **lactose fed-batch** | **whey fed-batch** |
| pyruvate | 5.75 ± 0.27 | 3.80 ± 2.32 | 6.36 ± 0.70 |
| acetate | 6.57 ± 0.12 | 3.62 ± 0.84 | 8.24 ± 0.41 |
| succinate | 3.19 ± 0.08 | 0.51 ± 0.31 | 0.26 ± 0.12 |
| diacetyl | 1.02 ± 0.04 | 0.55 ± 0.10 | 0.95 ± 0.24 |
| acetoin | 2.22 ± 0.03 | 1.24 ± 0.28 | 1.40 ± 0.18 |
| isobutyraldehyde | 1.44 ± 0.03 | 0.77 ± 0.06 | 1.09 ± 0.17 |

**Table S5** Performance of *E. coli* W *∆ldhA ∆adhE ∆pta ∆frdA* IB4 for each batch in glucose pulsed fed-batch experiments: mean volumetric (r_P_) and specific (q_P_) isobutanol production, volumetric (r_S_) and specific (q_S_) glucose uptake rates, isobutanol (Y_P/S_) biomass (Y_X/S_) and CO_2_ (Y_CO2/S_) yield, total carbon recoveries and percentage of isobutanol stripped into the wash-bottle. Means and standards deviations are calculated from duplicate experiments

| **glucose** | **r_P_**  **[g l^-1^ h^-1^]** | **q_P_**  **[g g^-1^ h^-1^]** | **r_S_**  **[g l^-1^ h^-1^]** | **q_S_**  **[g g^-1^ h^-1^]** | **Y_P/S_**  **[Cmol Cmol^-1^]** | **Y_X/S_**  **[Cmol Cmol^-1^]** | **Y_CO2/S_**  **[Cmol Cmol^-1^]** | **C rec.**  **[%]** | **isob. stripped [%]** |
| --- | --- | --- | --- | --- | --- | --- | --- | --- | --- |
| Batch 1 | 0.21  ± 0.01 | 0.10  ± 0.01 | 1.70  ± 0.04 | 0.76  ± 0.03 | 0.20 ±  0.01 | 0.085 ± 0.009 | 0.33 ±  0.02 | 79 ±  2 | 5  ± 1 |
| Batch 2 | 0.25  ± 0.01 | 0.06  ± 0.01 | 1.52  ± 0.04 | 0.40  ± 0.02 | 0.26 ±  0.01 | 0.016 ± 0.002 | 0.61 ±  0.05 | 89 ±  2 | 68  ± 1 |
| Batch 3 | 0.07  ± 0.01 | 0.02  ± 0.01 | 0.35  ± 0.04 | 0.08  ± 0.01 | 0.34 ±  0.08 | 0.039 ± 0.006 | 0.58 ± 0.09 | 102 ±  6 | 100  ± 1 |

**Table S6** Performance of *E. coli* W *∆ldhA ∆adhE ∆pta ∆frdA* IB4 for each batch in lactose pulsed fed-batch experiments: mean volumetric (r_P_) and specific (q_P_) isobutanol production, volumetric (r_S_) and specific (q_S_) glucose uptake rates, isobutanol (Y_P/S_) biomass (Y_X/S_) and CO_2_ (Y_CO2/S_) yield, total carbon recoveries and percentage of isobutanol stripped into the wash-bottle. Means and standards deviations are calculated from duplicate experiments. As one cultivation failed after batch 2, the parameters of batch 3 and for the total batch are calculated from one replicate

| **lactose** | **r_P_**  **[g l^-1^ h^-1^]** | **q_P_**  **[g g^-1^ h^-1^]** | **r_S_**  **[g l^-1^ h^-1^]** | **q_S_**  **[g g^-1^ h^-1^]** | **Y_P/S_**  **[Cmol Cmol^-1^]** | **Y_X/S_**  **[Cmol Cmol^-1^]** | **Y_CO2/S_**  **[Cmol Cmol^-1^]** | **C rec.**  **[%]** | **isob. stripped [%]** |
| --- | --- | --- | --- | --- | --- | --- | --- | --- | --- |
| Batch 1 | 0.23  ± 0.04 | 0.11  ± 0.01 | 1.34  ± 0.27 | 0.65  ± 0.07 | 0.27 ± 0.01 | 0.088 ± 0.007 | 0.37 ± 0.02 | 94 ± 1 | 7  ± 1 |
| Batch 2 | 0.19  ± 0.01 | 0.06  ± 0.01 | 0.89  ± 0.02 | 0.26  ± 0.02 | 0.33 ± 0.01 | 0.029 ± 0.009 | 0.62 ± 0.01 | 93 ± 1 | 61  ± 1 |
| Batch 3 | 0.09 | 0.03 | 0.32 | 0.10 | 0.32 | 0.010 | 0.79 | 102 | 86 |

**Table S7** Performance of *E. coli* W *∆ldhA ∆adhE ∆pta ∆frdA* IB4 for each batch in cheese whey pulsed fed-batch experiments: Mean volumetric (r_P_) and specific (q_P_) isobutanol production, volumetric (r_S_) and specific (q_S_) lactose uptake rates and percentage of isobutanol stripped into the wash-bottle. Means and standards deviations are calculated from duplicate experiments

| **whey** | **r_P_**  **[g l^-1^ h^-1^]** | **q_P_**  **[g g^-1^ h^-1^]** | **r_S_**  **[g l^-1^ h^-1^]** | **q_S_**  **[g g^-1^ h^-1^]** | **isob. stripped [%]** |
| --- | --- | --- | --- | --- | --- |
| Batch 1 | 0.21  ± 0.01 | 0.08  ± 0.01 | 1.41  ± 0.01 | 0.57  ± 0.06 | 5  ± 1 |
| Batch 2 | 0.29  ± 0.01 | 0.08  ± 0.01 | 1.97  ± 0.05 | 0.51  ± 0.02 | 43  ± 1 |
| Batch 3 | 0.24  ± 0.03 | 0.05  ± 0.01 | 1.34  ± 0.17 | 0.30  ± 0.03 | 55  ± 1 |
| Batch 4 | 0.18  ± 0.05 | 0.04  ± 0.01 | 1.00  ± 0.11 | 0.20  ± 0.02 | 72  ± 1 |
| Batch 5 | 0.12  ± 0.04 | 0.02  ± 0.01 | 1.17  ± 0.13 | 0.22  ± 0.02 | 87  ± 3 |

**Table S8** Performance of *E. coli* W *∆ldhA ∆adhE ∆pta ∆frdA* IB4 for each batch in cheese whey pulsed fed-batch experiment: Mean isobutanol (Y_P/S_), biomass (Y_X/S_), isobutanol (Y_P/S_) and CO_2_ (Y_CO2/S_) yield and total carbon recoveries. Yields were calculated considering lactose (S1) or the sum of lactose and lactate (S2) as substrates. Means and standards deviations are calculated from duplicate experiments

| **whey** | **Y_P/S1_**  **[Cmol Cmol^-1^]** | **Y_P/S2_**  **[Cmol Cmol^-1^]** | **Y_X/S1_**  **[Cmol Cmol^-1^]** | **Y_X/S2_**  **[Cmol Cmol^-1^]** | **Y_CO2/S1_**  **[Cmol Cmol^-1^]** | **Y_CO2/S2_**  **[Cmol Cmol^-1^]** | **C rec.**  **[%]** |
| --- | --- | --- | --- | --- | --- | --- | --- |
| Batch 1 | 0.23  ± 0.01 | 0.21  ± 0.01 | 0.11  ± 0.02 | 0.10  ± 0.01 | 0.35  ± 0.03 | 0.32  ± 0.03 | 93  ± 8 |
| Batch 2 | 0.29  ± 0.01 | 0.26  ± 0.01 | 0.05  ± 0.01 | 0.04  ± 0.01 | 0.55  ± 0.06 | 0.51  ± 0.06 | 94  ± 8 |
| Batch 3 | 0.28  ± 0.07 | 0.24  ± 0.05 | 0.07  ± 0.01 | 0.06  ± 0.01 | 0.61  ± 0.19 | 0.52  ± 0.15 | 100  ± 7 |
| Batch 4 | 0.29  ± 0.11 | 0.25  ± 0.10 | 0.04  ± 0.03 | 0.03  ± 0.02 | 0.73  ± 0.16 | 0.64  ± 0.13 | 90  ± 3 |
| Batch 5 | 0.17  ± 0.07 | 0.16  ± 0.07 | 0.02  ± 0.04 | 0.02  ± 0.01 | 0.69  ± 0.14 | 0.65  ± 0.13 | 90  ± 1.5 |
